# Diffusion Weighted and Diffusion Tensor Imaging: A Clinical Guide

**DOI:** 10.1055/s-0038-1669394

**Published:** 2018-08-13

**Authors:** J. Bahm

**Affiliations:** 1 Euregio Reconstructive Microsurgery Unit, Franziskus Hospital, Aachen, Germany

**Diffusion Weighted and Diffusion Tensor Imaging: A Clinical Guide edited by C. da Costa Leite and M. Castillo [Thieme 2016]**

Imaging of nerve tissue morphology progressed significantly with the development of magnetic resonance imaging (MRI) technology. A step further is done actually with the diffusion tensor imaging (DTI) applied on MRI signals, where one can visualize not only white matter bundles, but also starts to get insight in physiologic processes like brain maturation and nerve regeneration) as well as in pathologies like tumors, infectious diseases, demyelination, injury, and hemorrhage.

This is a novel up to date book in a new and thrilling clinical and research field, edited and written by neuroradiologic experts.

After a comprehensive review of the underlying physics and the anatomy of supratentorial white matter tracts and their organization, the editors present chapters about imaging of the brain during the first 2 years of life (development and aging changes), before addressing the aforementioned main fields of brain pathology—not without dedicating a separate chapter to the spine and spinal cord diseases.

The last chapter even goes beyond the future and deals with even newer developments based on non-Gaussian signal distribution, introducing the research on diffusional kurtosis and diffusion spectrum imaging and their potential applications, trying to improve the representation of crossing axonal fibers and tracts, a serious limitation of DTI images.

Every chapter is written very clearly and has a well-defined structure, with beautiful illustrations in order to capture the focus of non-radiologic readers within the new field of imaging and research. Of course, a better visualization of organized tracts and their alteration in pathology and aging will stimulate our pathophysiologic curiosity and drive them to investigate further.

For neurologists, potentially hypothetic tissue changes such as in early Alzheimer's disease now become obvious. For neurosurgeons who deal with a vascular pathology or a tumor, the alteration of neighboring tracts is precisely represented. The peripheral nerve surgeon starts to follow in the postoperative course regenerating cones through the morphologic highway of a peripheral nerve structure, recognizable by their clear unidirectional fluid and tissue movement, so precisely identifiable on tractography.

DTI is not necessarily expensive, but time consuming. Thus far, it is not a routine procedure either in neuroradiology investigation or in medical cost reimbursement considerations.

This book provides quick updates to a lot of concerned physicians and technicians.

